# An Exploratory Study on the Efficacy of a New Heat and Moisture Exchanger (Provox® LifeTM) for Laryngectomized Patients

**DOI:** 10.7759/cureus.73279

**Published:** 2024-11-08

**Authors:** Hiroko Takeda, Hirotaka Shinomiya, Hajime Fujiwara, Tatsuya Furukawa, Masanori Teshima, Ken-ichi Nibu

**Affiliations:** 1 Department of Otolaryngology-Head and Neck Surgery, Kobe University Hospital, Kobe, JPN

**Keywords:** adhesive, artificial nose, heat and moisture exchanger, laryngectomy, respiratory symptoms

## Abstract

Objective: After a total laryngectomy, patients lose various physiological functions of the upper airway. To address these issues, an artificial nose (heat and moisture exchanger (HME)) has been developed. However, the current HMEs do not provide sufficient physiological upper airway function. We prospectively investigated the efficacy of a new type of HME and compared it with that of the current type.

Methods: This was a pre- and post-comparative study comparing the efficacy of the current HME and the new HME using a questionnaire (physiological upper airway function, skin complications, and adherence, especially for nocturnal HME). Patients who underwent laryngectomy were asked to use the new type of HME for six weeks and to complete questionnaires three days before and six weeks after starting the use of the new HME. The values for each question were statistically compared using paired *t*-tests.

Results: A significant decrease in the number of coughs per day was observed (before use: 6.89, after use: 3.16; p = 0.049). The degree of dyspnea significantly improved (before use: 7.77 cm, after use: 9.28 cm; p = 0.013). The number of days of nocturnal HME use improved significantly from 0 days to 33.32/42 days after the use of the new HME (p <0.0001).

Conclusion: To the best of our knowledge, for the first time in Japan, we showed that the new HME can improve respiratory symptoms, such as the number of coughs per day, degree of breathlessness, and nocturnal HME use.

## Introduction

Total laryngectomy is a well-established radical surgical treatment for locally advanced laryngeal, hypopharyngeal, and cervical esophageal cancers and has been performed in various institutions. After total laryngectomy, patients lose physiological upper airway functions, including heating, humidification, and filtration, owing to the diversion of the upper and lower airways [1]. Air is taken directly through a permanent tracheostoma, increasing airway secretions and respiratory infections, which cause a range of problems in daily life [2]. To address these issues, an artificial nose (heat and moisture exchanger (HME)) has been developed. Heat and moisture exchangers (Provox® Vega™ series: XtraMoist™ and XtraFlow™) are now covered by public insurance in Japan [3,4]. However, the current HMEs do not provide sufficient physiological upper airway function. Thus, patients using HMEs still suffer from respiratory symptoms such as coughing and increased sputum production [5]. Furthermore, adverse events such as skin disorders due to adhesives may occur, leading to reduced adherence [6]. Nocturnal HMEs, which have a greater humidifying effect than daytime HMEs, are not widely used in Japan.

To solve these problems, a new type of HME (Provox Life™, Atos Medical, Sweden) has been developed. The diameter of the new HME is 1 mm larger than that of the current HME, resulting in a 15.5% improvement in aerodynamic resistance and humidification. The adhesive material and structure of the connection between the adhesive and the HME have also been improved to reduce skin irritation and the coming off of the HME come off during coughing and talking. Additionally, while a typical HME requires different adhesives for daytime and nighttime use, the new type of HME allows the use of the same adhesive. Therefore, adherence to nocturnal HME may improve. In the present study, we prospectively investigated the efficacy of a new type of HME compared with the current type from the viewpoints of physiological upper airway function, skin complications, and adherence, especially nocturnal HME.

## Materials and methods

Patients and methods

Subjects and Setting

The study was conducted between July and December 2023. Patients who underwent laryngectomy at our hospital and had been using HME for at least three months were included in this study. Written consent was obtained from all patients. The selection and exclusion criteria are listed in Table 1.

**Table 1:** Inclusion and exclusion criteria HME: heat and moisture exchanger

Inclusion criteria	Exclusion criteria
Age ≧18 years old	Unable to put on or take off HME by oneself
After total laryngectomy	There are metastases or recurrences.
＞1 year after total laryngectomy	There is a skin disorder that requires treatment at the site where the HME is attached.
>6 weeks after postoperative radiotherapy	Unable to understand the questionnaire or consent form due to cognitive decline, etc.
>3 months after starting to use HME	<1 month after the onset of a pulmonary infection or exacerbation of a chronic respiratory disease
Clinically determined to be able to use the Provox® LifeTM Home HME	<1 month after changing the dosage of cough medicine or expectorant
Written consent is obtained from the patient.	The principal investigator judged it to be inappropriate.

Study Design

**Figure 1:**
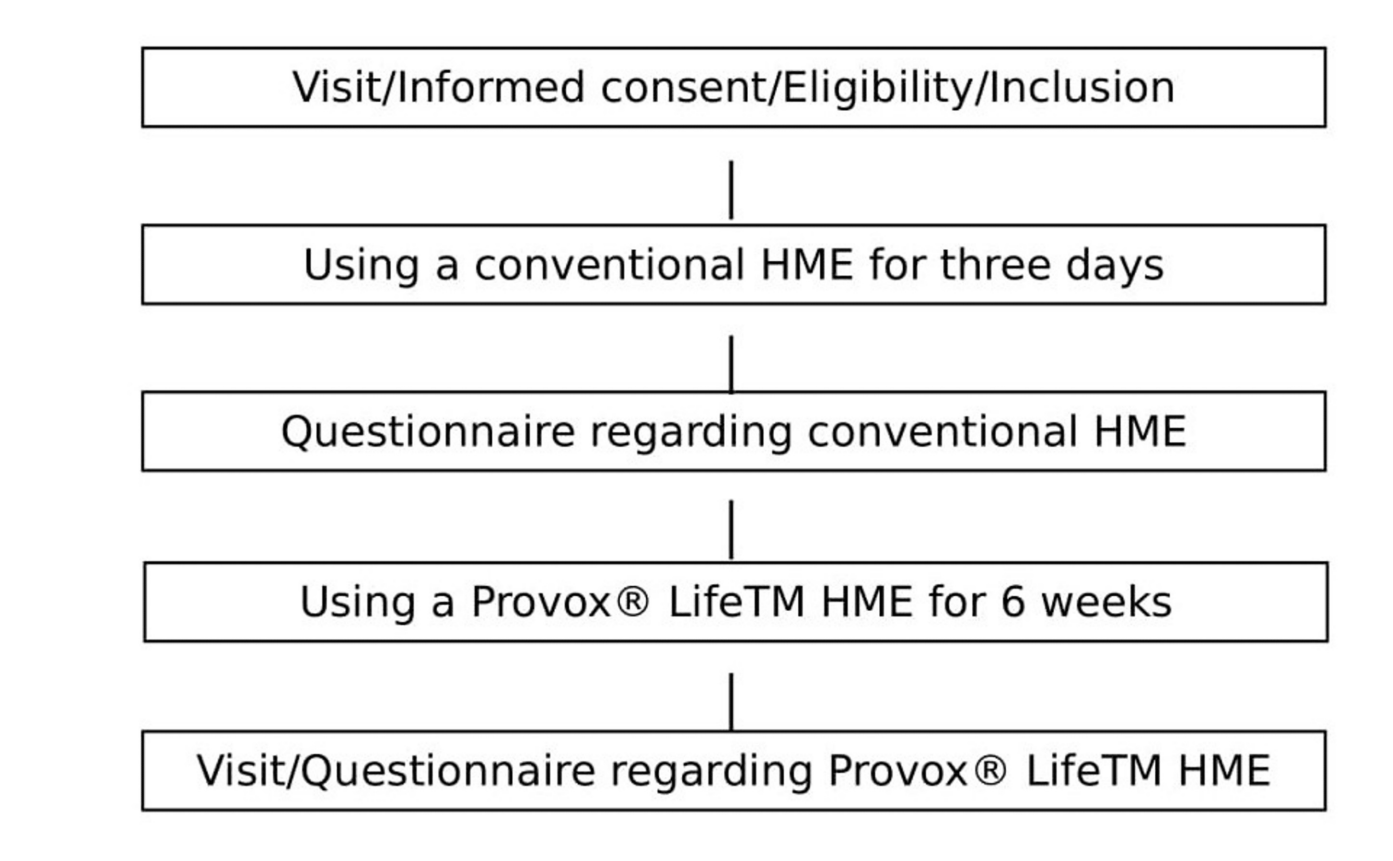
Flow diagram of the study steps Patients were asked to use the new type of HME for six weeks and complete questionnaires three days before and six weeks after starting its use. HME: heat and moisture exchanger

Longobardi et al. [7] reported that the number of coughs per day was 6.7 in the current HME group and 4.3 in the new HME group (P = 0.0001). Assuming that a similar result would be obtained in this study, we set the alpha (α) error at 5% and the beta (β) error at 20% and determined the number of patients to be approximately 20. This was a pre- and post-comparative study comparing the efficacy of current and new HMEs using a questionnaire. A flowchart of the study is shown in Figure 1.

Patients were asked to use the new type of HME for six weeks and complete questionnaires three days before and six weeks after starting its use. The values for each question were statistically compared using a paired t-test in Microsoft Excel 2019 for Windows (Microsoft Corp., Redmond, WA). All t-tests were two-tailed and were assessed at a significance level of 5%. This study was approved by the Ethics Committee of Kobe University Hospital (ethics code: A230002).

Materials

**Figure 2:**
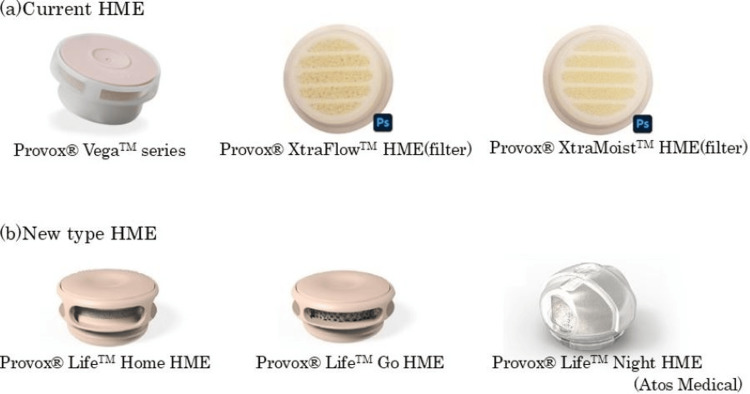
Comparison of current and new type HME Provox® LifeTM Go HME has low respiratory resistance and is suitable for daytime activity, similar to Provox® XtraFlowTM HME. Provox® LifeTM Home HME has excellent humidification capability and is suitable for daytime rest, similar to Provox® XtraMoistTM HME. Provox® LifeTM Night HME is a nocturnal HME, which has a high humidification capacity and is made of soft material so that turning over in bed does not cause pain. The diameter of the new type of HME is 1 mm larger than that of the current type, resulting in a 15.5% improvement in aerodynamic resistance and humidification. HME: heat and moisture exchanger

“Home,” “Go,” and “Night” of Provox® Life™ series (Atos Medical, Sweden) were used in this study (Figure 2).

“Home” has excellent humidification capability and is suitable for daytime rest; “Go” has low respiratory resistance and is suitable for daytime activity; and “Night” is a nocturnal HME with high humidification capacity and is made of a soft material to prevent pain when turning over in bed [8]. In this study, patients who had been using Provox® XtraMoistTM HME prior to this study were assigned to use “Home,” while patients who had been using Provox® XtraFlowTM HME were assigned to use “Go” for six weeks. “Night” was used for all patients. All HMEs and adhesives were provided free of charge. No other financial incentives were offered.

Assessment Items

The questionnaire was based on the Cough and Sputum Assessment Questionnaire (CASA-Q) [9]. The primary endpoint was the number of coughs per day. If a cough occurs repeatedly, it is counted as one cough. Coughs that occur within 10 seconds of the previous cough are counted as repeated coughs. Secondary endpoints were 1) the number of coughs within 30 min of waking up, 2) number of awakenings due to coughing during sleep, 3) number of (forced) sputum expectoration, 4) number of occurrences of colored and bloody sputum, 5) number of times a sputum aspirator was used, 6) ease of expectorating, 7) frequency and ease of cleaning permanent tracheostoma, 8) presence of skin disorders such as bleeding from a permanent tracheostomas, 9) wearing time, 10) degree of dyspnea, 11) number of days of nocturnal HME use, 12) ease of wearing and removing, and 13) satisfaction with the new HME. For all assessment items, the values three days before wearing the new HME were statistically compared with the values three days before the end of wearing the new HME. The number of times per day was assessed for items related to frequency. Items related to the scale were rated on a Visual Analog Scale (VAS), with “very bad” at the left end of a 10 cm straight line and “very good” at the right end. Patients were asked to mark the line at the appropriate position, and the distance (cm) from the left end to the marked line was measured. The higher the number, the better the evaluation.

## Results

Patient Characteristics

Twenty patients (19 males and one female) were included in the study based on the inclusion and exclusion criteria. At the start of the study, one patient out of 20 refused to use the new HME and was excluded. Detailed patient characteristics are shown in Table 2.

**Table 2:** Patient characteristics HME: heat and moisture exchanger; -: not applicable

Characteristics	No./ Mean±SD	Percentage (%)	Range
Age(years)	70.8±7.53	-	53-81
Sex			
Male	19	95	-
Female	1	5	-
Primary tumor location			
Larynx	7	35	-
Hypopharynx	11	55	-
Cervical esophagus	2	10	-
Months since using HME	31.2±22.86	-	3-89
Types of HME			
Go	14	70	-
Home	6	30	-
Provox			
Yes	7	35	-
No	13	65	-
Brinkman Index (Average)	708.5±342.51	-	0-1600

Questionnaire

**Figure 3:**
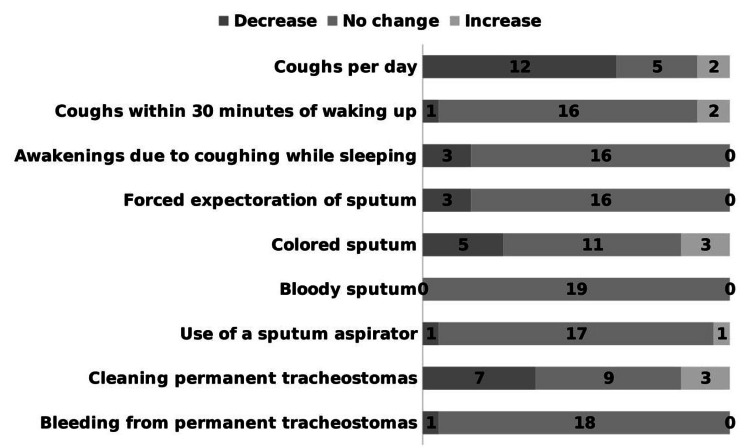
Frequency evaluation items percentages For each frequency item, the responses are grouped according to whether they have decreased, not changed, or increased after the use of the new HME compared to before. For all items, there are many groups with no changes. There is a decreasing trend in the number of coughs per day, number of coughs during sleep, number of (forced) expectorations of sputum, number of occurrences of colored sputum, and frequency of permanent tracheostoma cleaning. HME: heat and moisture exchanger

The results of the questionnaire are presented in Table 3. In the frequency assessment items, most patients answered 0 times, and a few patients answered a large number of times. As a result, the variability increased, and in some cases, the SD values exceeded the mean values. Regarding the frequency assessment items, a significant decrease was observed in the number of coughs per day (before: 6.89, after: 3.16; p = 0.049). Although the statistical analyses did not reach significance, there was a decreasing trend in the number of coughs during sleep, number of (forced) expectorations of sputum, number of occurrences of colored sputum, and frequency of cleaning the permanent tracheostomy (Figure 3).

**Table 3:** Questionnaire results HME: heat and moisture exchanger; -: No p-value because ‘before’ and ‘after’ are both 0; *: significance level of p < 0.05 (a) shows the results of the frequency items; (b) shows the results of the scale items; (c) shows the results of HME use items

(a) Frequency items (times/day)	Before (Mean±SD)	After (Mean±SD)	p
Coughs per day	6.89±6.63	3.16±4.09	0.049*
Coughs within 30 minutes of waking up	0.32±0.65	0.42±0.82	0.193
Awakenings due to coughing while sleeping	0.21±0.52	0	0.095
Forced expectoration of sputum	0.58±1.46	0.05±0.22	0.14
Colored sputum	3.21±5.57	1.74±3.54	0.35
Bloody sputum	0	0	-
Use of a sputum aspirator	0.21±0.69	0.16±0.49	0.794
Cleaning permanent tracheostomas	5.92±8.59	4.53±6.15	0.579
Bleeding from permanent tracheostomas	0.11±0.31	0.05±0.22	0.56
(b) Scale items (cm/10cm)	Before (Mean±SD)	After (Mean±SD)	p
Ease of coughing up sputum	7.95±2.71	8.35±2.11	0.62
Ease of cleaning permanent tracheostomas	7.34±2.73	7.552.90	0.824
Degree of dyspnea	7.77±2.09	9.28±1.28	0.013*
Ease of wearing and removing	8.89±2.31	9.21±1.61	0.637
Satisfaction with the HME	7.33±2.40	7.87±2.10	0.48
(c) HME use	Before (Mean±SD)	After (Mean±SD)	p
Days of nocturnal HME worn (/42 days)	0	33.32±11.58	2.3×10-^14^*
Wearing time (hours)	23.79±0.89	23.84±0.66	0.843

**Figure 4:**
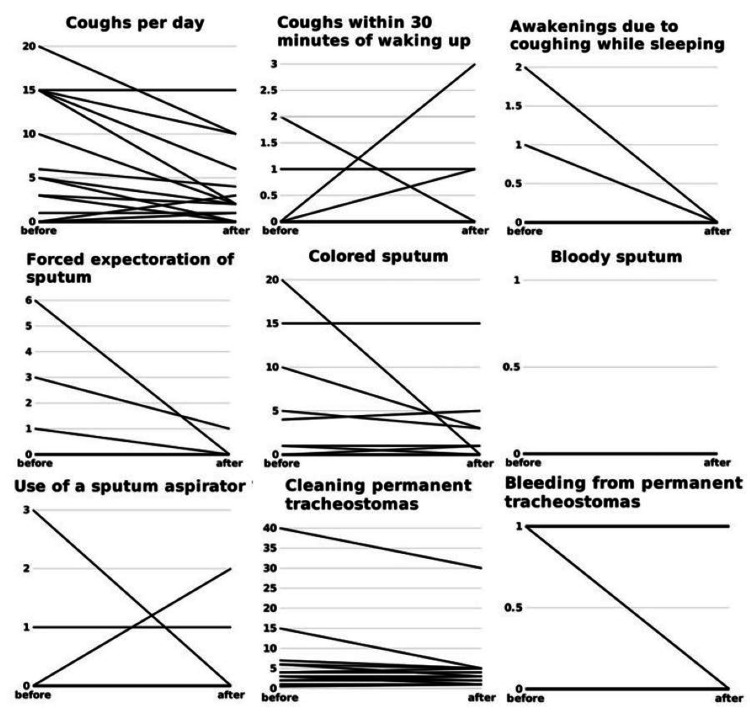
Scores of frequency evaluation items In the frequency assessment items, most patients answered 0 times both before and after using the new HME. A few patients answered a large number of times. Although the statistical analyses did not reach significance, there is a decreasing trend in the number of coughs during sleep, number of (forced) expectorations of sputum, number of occurrences of colored sputum, and frequency of cleaning the permanent tracheostoma. This is particularly noticeable in patients with high frequency before use. HME: heat and moisture exchanger

This was particularly noticeable in patients with a high frequency before use (Figure 4).

Among the scale assessment items, the degree of dyspnea significantly improved (before use: 7.77 cm, after use: 9.28 cm; p = 0.013). The number of days of nocturnal HME use improved significantly from 0 to 33.32/42 after using the new HME (p = 2.3 × 10-14). No new skin disorders caused by HME were observed. In the free-text sections, the patients stated, “breathing easier,” “easier to put on and take off,” “very satisfied with the daytime HME, but the nocturnal HME comes off easily,” and “the nighttime version came off several times while I was sleeping.”

## Discussion

Patients who undergo total laryngectomy lose physiological upper airway functions such as warming, humidification, and filtration [1]. Histological changes in the respiratory epithelium, such as impaired mucociliary clearance of the lower airway and loss of ciliated cells, have been reported in laryngectomized patients, leading to increased airway secretion and respiratory infection [10]. Because these respiratory symptoms impair social contact and mental health and cause chronic fatigue, sleep disturbances, and depressive symptoms [11], airway management in the early postoperative period is crucial. Compensatory upper airway function with HME has been reported to restore ciliated cells in the lower airway [10], leading to reduced cough and sputum production and improved quality of life [3,12].

In this study, we prospectively investigated the effectiveness of a new HME, which was developed to improve humidification and airway resistance, compared with the current HME using a questionnaire. Similar studies conducted in Italy and Australia reported significant improvements in daily cough frequency, forced expectoration of sputum, and breathlessness after the use of a new HME [7,13]. Since the Japanese climate is warm and humid, especially in our area, we believed that the effectiveness of the new type of HME would vary according to race and climate. However, in this study, the number of coughs per day and breathlessness significantly improved, in accordance with previous studies. To the best of our knowledge, this is the first report in Japan showing that the use of a new HME can improve respiratory symptoms.

Since the adhesive material has also been improved to reduce skin irritation, a decrease in skin disorders was expected. In this study, there was no significant reduction in skin disorders because there were fewer skin disorders before using the new HME.

Additionally, although the statistical analyses did not reach significance, the number of coughs during sleep, sputum expectoration, occurrences of colored sputum, and frequency of cleaning permanent tracheostomas decreased in patients with high scores for each symptom before using the new HME (Figure 4). As this study included patients who had been using the standard HME for more than three months, many patients scored zero for each symptom, both before and after using the new HME. This may explain why no significant differences were observed between groups. These findings suggest that HME improves respiratory symptoms immediately after laryngectomy.

Furthermore, nocturnal HME use increased significantly from 0 days to 33.32/42 days with the new HME. Longobardi et al. also reported a low rate of nocturnal HME use (12.5%) with the current HME, which increased to 80% with the use of new HMEs [7]. The new HMEs share the same adhesives for daytime and nocturnal HME, reducing the incidence of skin disorders as patients do not need to change their adhesives. This might explain the increased use of nocturnal HME. The use of nocturnal HME has been reported to reduce the number of awakenings due to coughing and increase the frequency of comfortable night sleep, although the difference was not statistically significant [14]. Similarly, the number of coughs decreased. Taken together, these findings suggest that increased adherence to nocturnal HME improves the patients’ quality of life.

Respiratory symptoms in patients who have undergone laryngectomy change seasonally, worsening during cold and dry weather [15,16]. Therefore, previous studies have been conducted in winter [7,15]. Although this study was conducted mainly in summer and autumn, we found that respiratory symptoms improved with the use of the new HME. Furthermore, all participants in this study had been wearing a daytime HME for at least three months (24 hours a day) before participating in this study. Since respiratory symptoms began to improve after approximately two weeks of wearing the HME [17], participants in this study were considered to have fully benefited from the standard type of HME. Thus, the present study demonstrated that, even in patients whose respiratory symptoms were already stable with the use of a standard HME, the use of a new HME could further improve respiratory symptoms.

This study had several limitations. Although the study design was a before-and-after comparative study, a randomized controlled trial would be better for a rigorous evaluation of the efficacy of the new HME. Because the number of laryngectomy patients attending our hospital was limited, it was difficult to enroll a sufficient number of patients to divide them into two groups. Therefore, we conducted a before-and-after comparative study.

In this study, all information regarding efficacy was based on subjective patient reports. Patients may have subconsciously reported better results because of the preconception that they were using improved HMEs. These results may be affected by placebo effects, patient expectations, and recall bias. In the future, objective assessments, such as the histological evaluation of bronchial epithelial cells, should be considered. The number of times the questionnaire is completed could also be a limitation. Increasing the number of times the questionnaire is completed could make it possible to evaluate cough improvement in more detail. In this study, the questionnaire was completed only twice in order to reduce the patient's burden. In the frequency assessment items, most patients answered 0 times, and a few patients answered a large number of times. As a result, the variability increased, and there is a risk of a decrease in statistical reliability.

Additionally, there is a possibility of selection bias among the participants. Most participants were cooperative and proactive due to the characteristics of this study, which may result in a positive bias in the number of days and hours of new HME use. Also, the patients had not used nocturnal HME before this study. Therefore, it is possible that the use of nocturnal HME improved the respiratory symptoms. In the future, a comparative study between current HME and new HME during the initial introduction phase following total laryngectomy should be considered.

The final limitation was the type of HME used. Previous studies have reported that high efficacy can be expected by using the various Provox® Life^TM^ HME series products (Home, Go, Energy, and Night) according to the situation [13]. However, the Japanese insurance system limits the number of HMEs used per month to 60, making it difficult to use HMEs in the same way as in previous studies. Therefore, this study was designed to use one type of HME for both daytime and nighttime.

## Conclusions

In conclusion, this study demonstrates that the HME significantly improves key respiratory symptoms for post-laryngectomy patients. Compared to the existing HME, the new device reduced daily cough frequency and eased breathlessness, reflecting a closer restoration of physiological airway function. Additionally, adherence to nocturnal HME use increased substantially, indicating greater comfort and suitability for continuous use. This evidence suggests that the new HME design offers enhanced support for respiratory function and quality of life, marking a meaningful advancement for individuals in Japan and potentially worldwide who undergo total laryngectomy.
